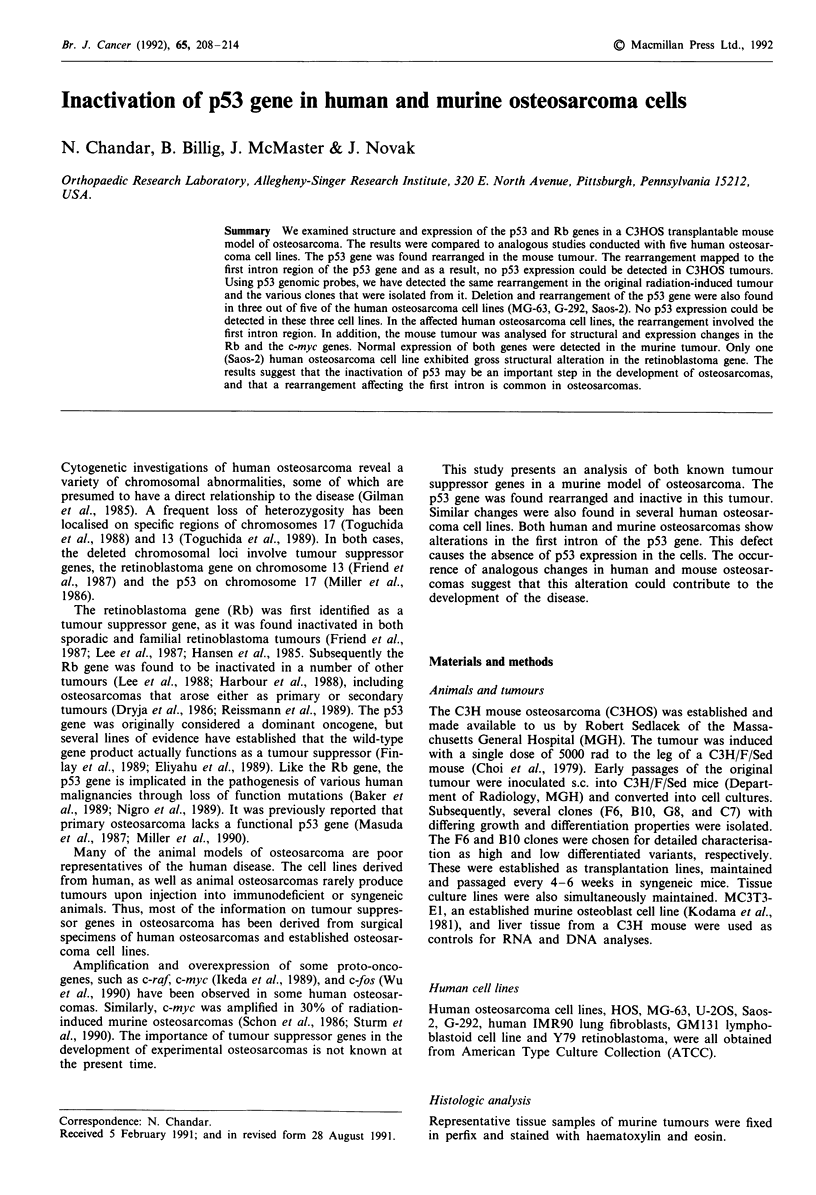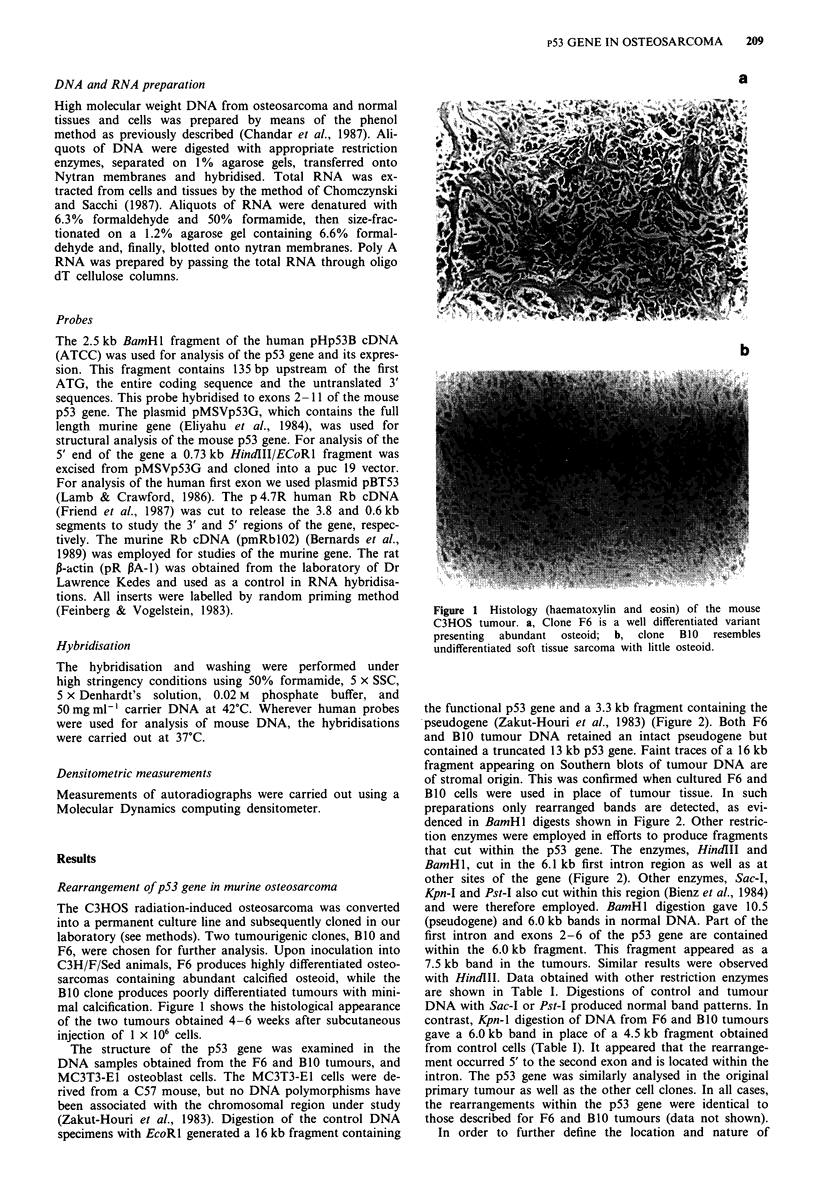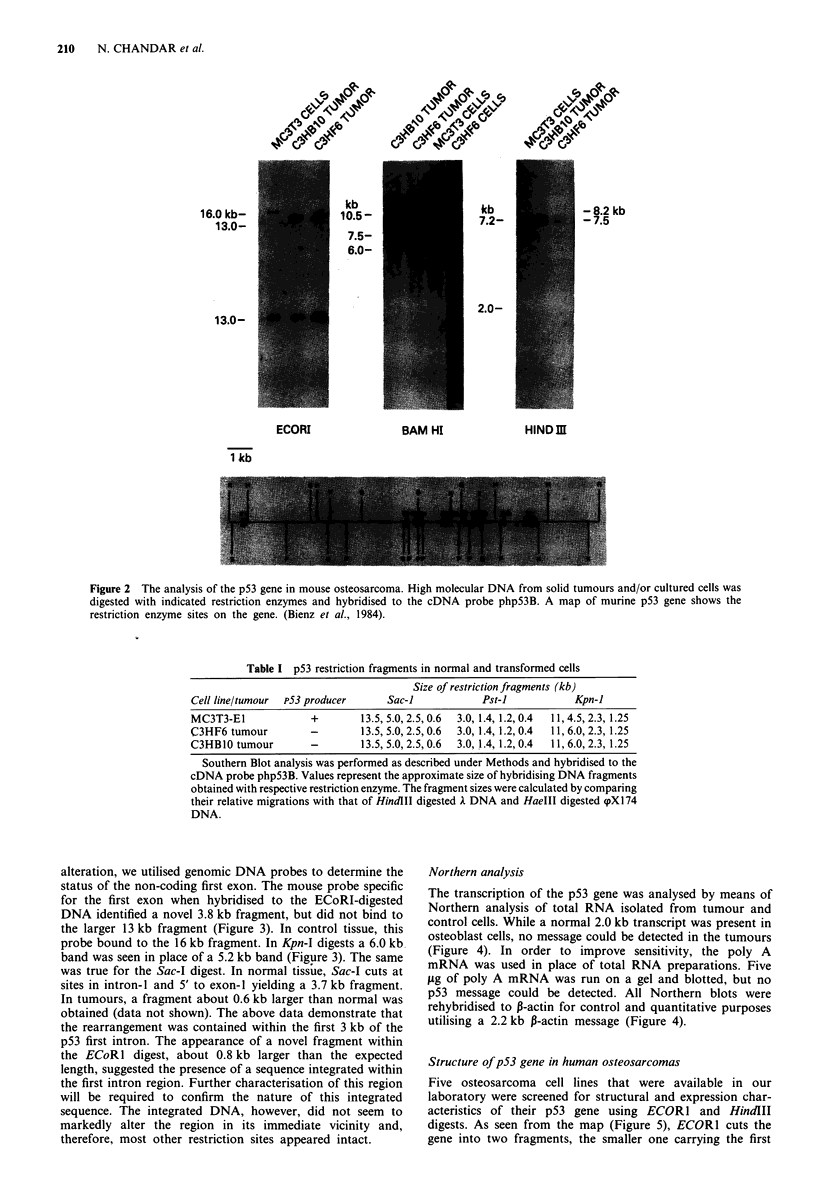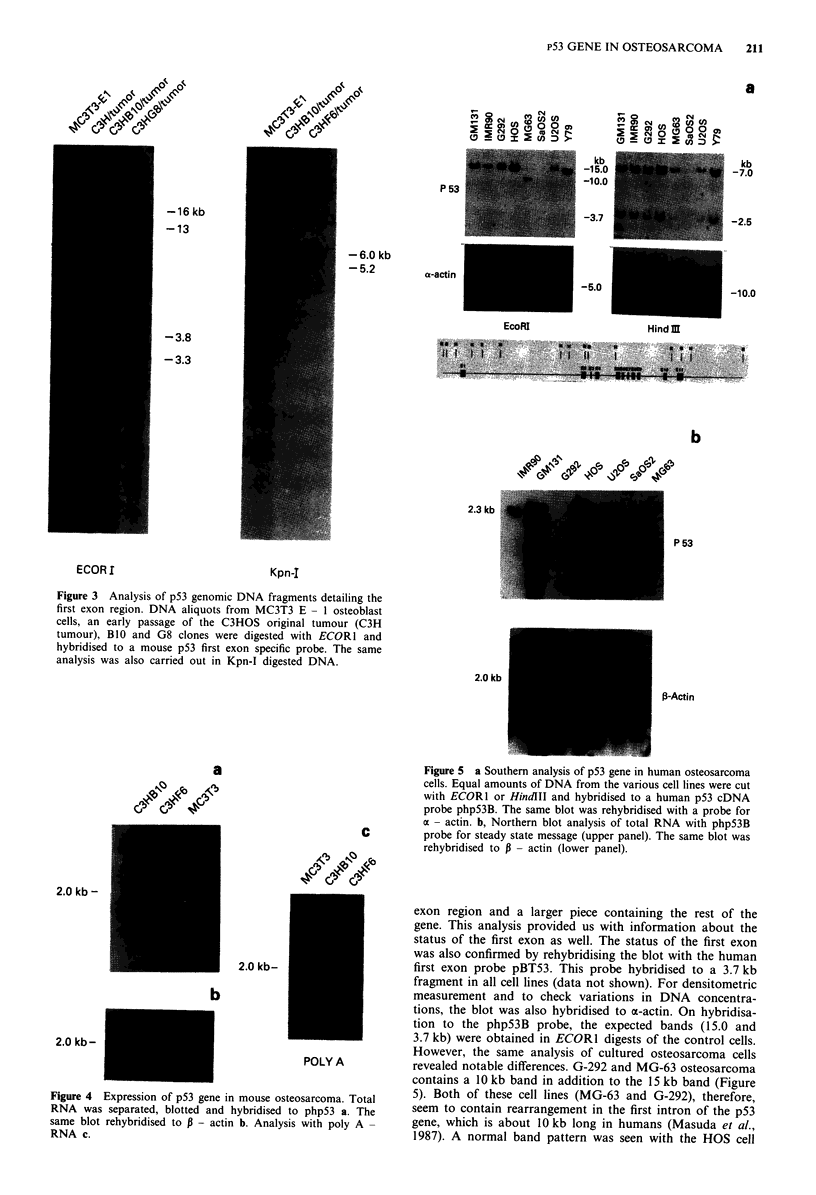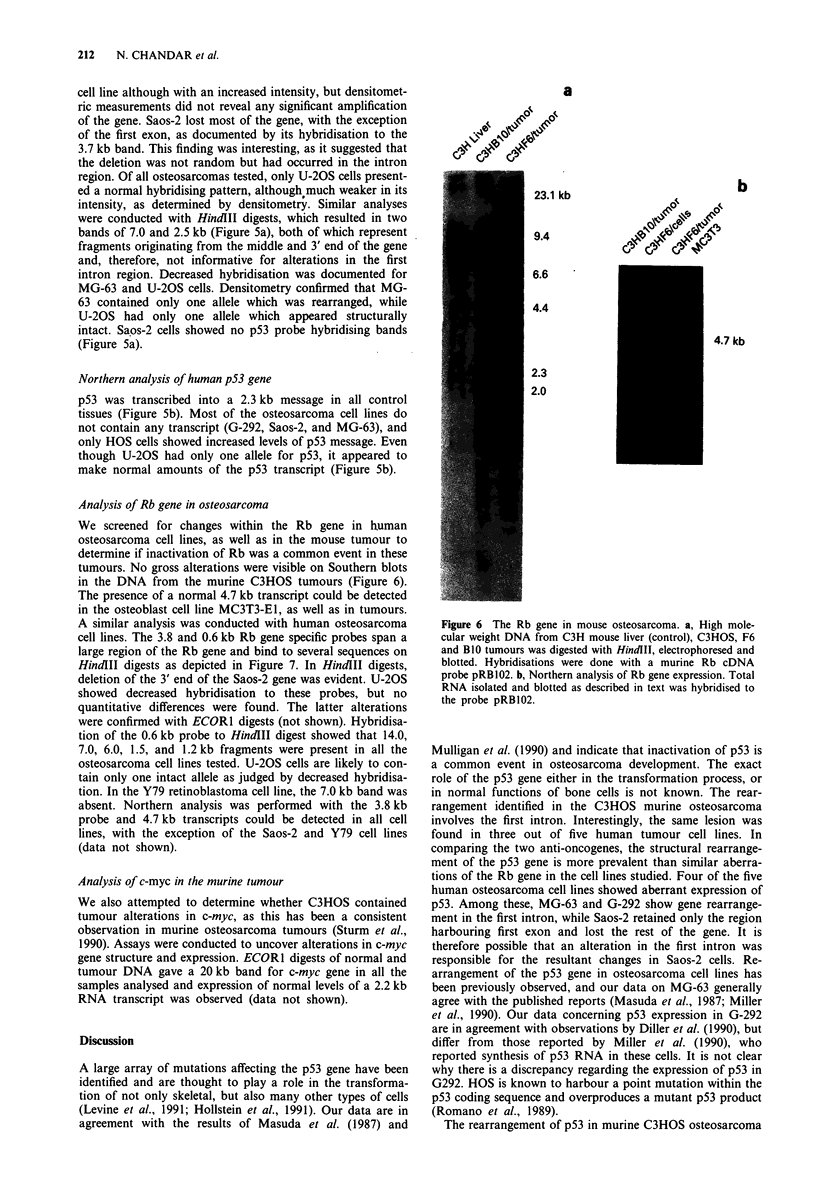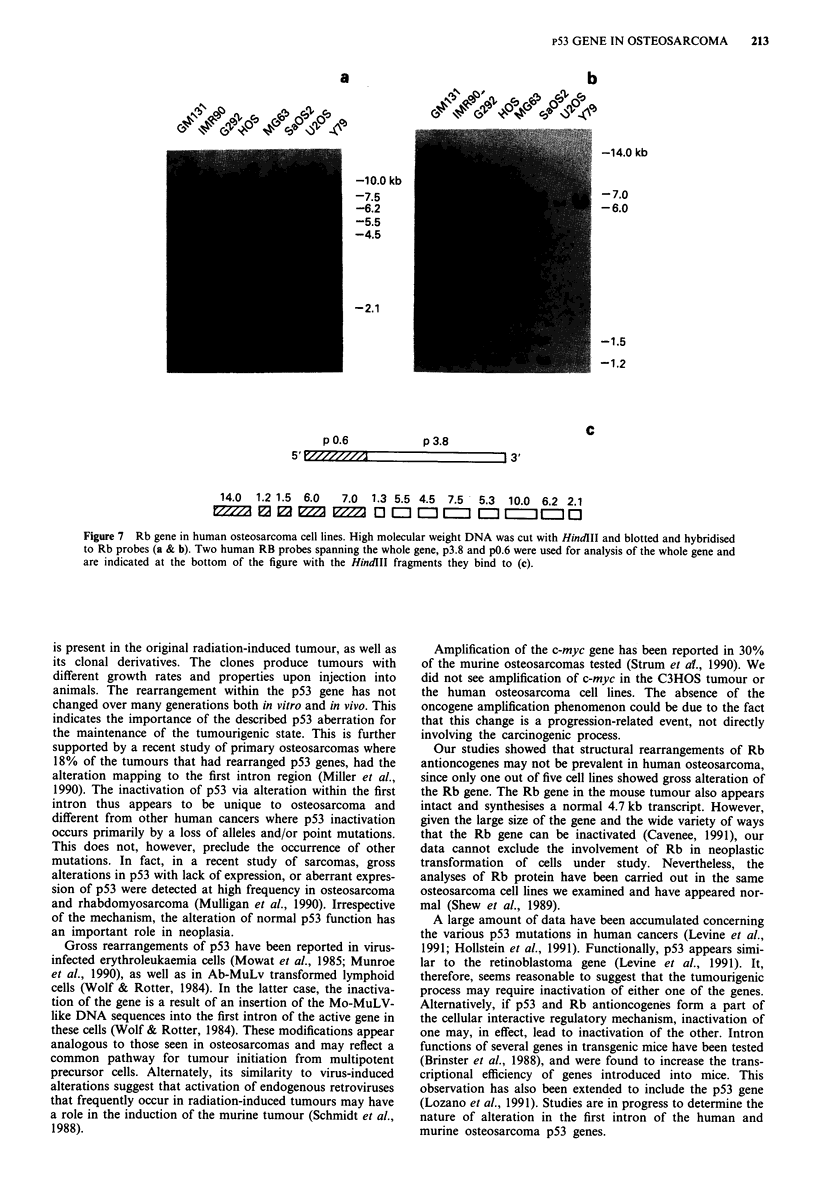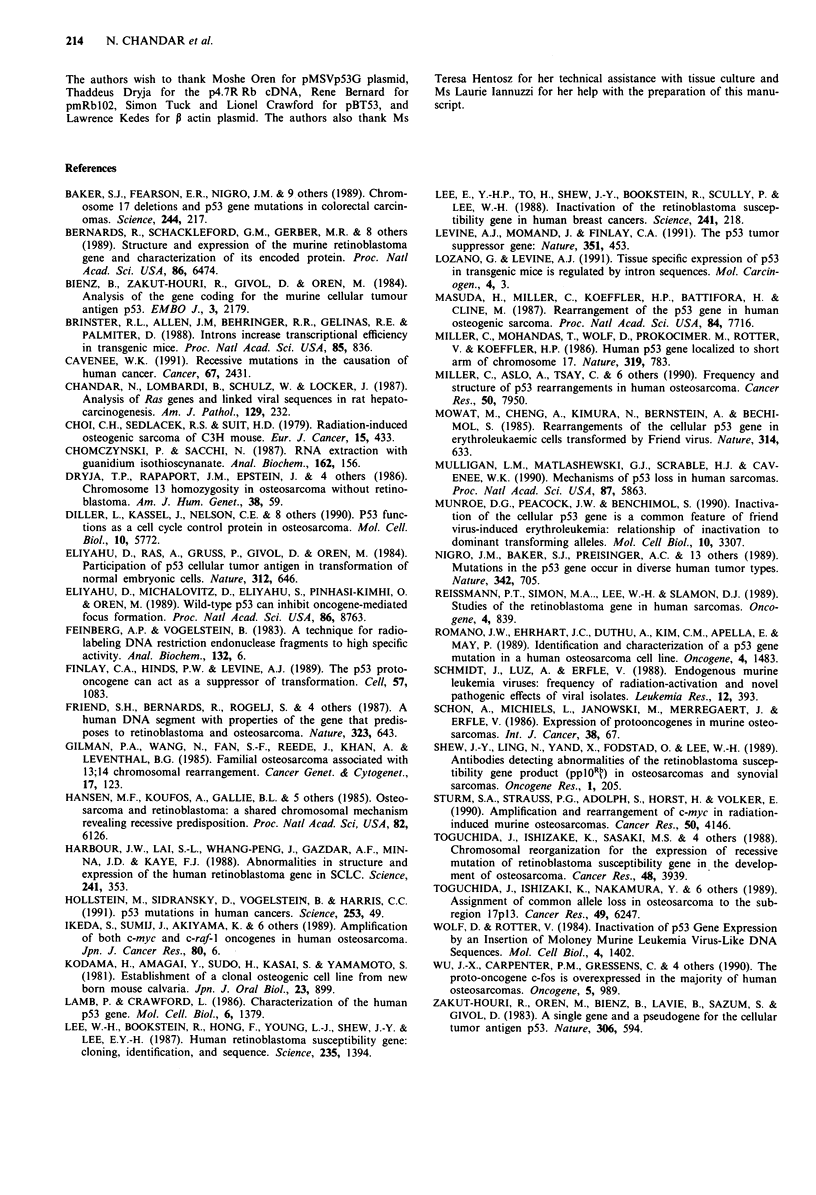# Inactivation of p53 gene in human and murine osteosarcoma cells.

**DOI:** 10.1038/bjc.1992.43

**Published:** 1992-02

**Authors:** N. Chandar, B. Billig, J. McMaster, J. Novak

**Affiliations:** Orthopaedic Research Laboratory, Allegheny-Singer Research Institute, Pittsburgh, Pennsylvania 15212.

## Abstract

**Images:**


*Br. J. Cancer* (1992), **65**, 208–214 © Macmillan Press Ltd., 1992

### Inactivation of p53 gene in human and murine osteosarcoma cells

N. Chandar, B. Billig, J. McMaster & J. Novak

*Orthopaedic Research Laboratory, Allegheny-Singer Research Institute, 320 E. North Avenue, Pittsburgh, Pennsylvania 15212, USA.*

**Summary** We examined structure and expression of the p53 and Rb genes in a C3HOS transplantable mouse model of osteosarcoma. The results were compared to analogous studies conducted with five human osteosarcoma cell lines. The p53 gene was found rearranged in the mouse tumour. The rearrangement mapped to the first intron region of the p53 gene and as a result, no p53 expression could be detected in C3HOS tumours. Using p53 genomic probes, we have detected the same rearrangement in the original radiation-induced tumour and the various clones that were isolated from it. Deletion and rearrangement of the p53 gene were also found in three out of five of the human osteosarcoma cell lines (MG-63, G-292, Saos-2). No p53 expression could be detected in these three cell lines. In the affected human osteosarcoma cell lines, the rearrangement involved the first intron region. In addition, the mouse tumour was analysed for structural and expression changes in the Rb and the c-*myc* genes. Normal expression of both genes were detected in the murine tumour. Only one (Saos-2) human osteosarcoma cell line exhibited gross structural alteration in the retinoblastoma gene. The results suggest that the inactivation of p53 may be an important step in the development of osteosarcomas, and that a rearrangement affecting the first intron is common in osteosarcomas.

Cytogenetic investigations of human osteosarcoma reveal a variety of chromosomal abnormalities, some of which are presumed to have a direct relationship to the disease (Gilman *et al.*, 1985). A frequent loss of heterozygosity has been localised on specific regions of chromosomes 17 (Toguchida *et al.*, 1988) and 13 (Toguchida *et al.*, 1989). In both cases, the deleted chromosomal loci involve tumour suppressor genes, the retinoblastoma gene on chromosome 13 (Friend *et al.*, 1987) and the p53 on chromosome 17 (Miller *et al.*, 1986).

The retinoblastoma gene (Rb) was first identified as a tumour suppressor gene, as it was found inactivated in both sporadic and familial retinoblastoma tumours (Friend *et al.*, 1987; Lee *et al.*, 1987; Hansen *et al.*, 1985. Subsequently the Rb gene was found to be inactivated in a number of other tumours (Lee *et al.*, 1988; Harbour *et al.*, 1988), including osteosarcomas that arose either as primary or secondary tumours (Dryja *et al.*, 1986; Reissmann *et al.*, 1989). The p53 gene was originally considered a dominant oncogene, but several lines of evidence have established that the wild-type gene product actually functions as a tumour suppressor (Finlay *et al.*, 1989; Eliyahu *et al.*, 1989). Like the Rb gene, the p53 gene is implicated in the pathogenesis of various human malignancies through loss of function mutations (Baker *et al.*, 1989; Nigro *et al.*, 1989). It was previously reported that primary osteosarcoma lacks a functional p53 gene (Masuda *et al.*, 1987; Miller *et al.*, 1990).

Many of the animal models of osteosarcoma are poor representatives of the human disease. The cell lines derived from human, as well as animal osteosarcomas rarely produce tumours upon injection into immunodeficient or syngeneic animals. Thus, most of the information on tumour suppressor genes in osteosarcoma has been derived from surgical specimens of human osteosarcomas and established osteosarcoma cell lines.

Amplification and overexpression of some proto-oncogenes, such as c-*raf*, c-*myc* (Ikeda *et al.*, 1989), and c-*fos* (Wu *et al.*, 1990) have been observed in some human osteosarcomas. Similarly, c-*myc* was amplified in 30% of radiation-induced murine osteosarcomas (Schon *et al.*, 1986; Sturm *et al.*, 1990). The importance of tumour suppressor genes in the development of experimental osteosarcomas is not known at the present time.

Correspondence: N. Chandar.
Received 5 February 1991; and in revised form 28 August 1991.

This study presents an analysis of both known tumour suppressor genes in a murine model of osteosarcoma. The p53 gene was found rearranged and inactive in this tumour. Similar changes were also found in several human osteosarcoma cell lines. Both human and murine osteosarcomas show alterations in the first intron of the p53 gene. This defect causes the absence of p53 expression in the cells. The occurrence of analogous changes in human and mouse osteosarcomas suggest that this alteration could contribute to the development of the disease.

### Materials and methods

### *Animals and tumours*

The C3H mouse osteosarcoma (C3HOS) was established and made available to us by Robert Sedlacek of the Massachusetts General Hospital (MGH). The tumour was induced with a single dose of 5000 rad to the leg of a C3H/F/Sed mouse (Choi *et al.*, 1979). Early passages of the original tumour were inoculated s.c. into C3H/F/Sed mice (Department of Radiology, MGH) and converted into cell cultures. Subsequently, several clones (F6, B10, G8, and C7) with differing growth and differentiation properties were isolated. The F6 and B10 clones were chosen for detailed characterisation as high and low differentiated variants, respectively. These were established as transplantation lines, maintained and passaged every 4–6 weeks in syngeneic mice. Tissue culture lines were also simultaneously maintained. MC3T3-E1, an established murine osteoblast cell line (Kodama *et al.*, 1981), and liver tissue from a C3H mouse were used as controls for RNA and DNA analyses.

### *Human cell lines*

Human osteosarcoma cell lines, HOS, MG-63, U-2OS, Saos-2, G-292, human IMR90 lung fibroblasts, GM131 lymphoblastoid cell line and Y79 retinoblastoma, were all obtained from American Type Culture Collection (ATCC).

### *Histologic analysis*

Representative tissue samples of murine tumours were fixed in perfix and stained with haematoxylin and eosin.

*DNA and RNA preparation*

High molecular weight DNA from osteosarcoma and normal tissues and cells was prepared by means of the phenol method as previously described (Chandar *et al.*, 1987). Aliquots of DNA were digested with appropriate restriction enzymes, separated on 1% agarose gels, transferred onto Nytran membranes and hybridised. Total RNA was extracted from cells and tissues by the method of Chomczynski and Sacchi (1987). Aliquots of RNA were denatured with 6.3% formaldehyde and 50% formamide, then size-fractionated on a 1.2% agarose gel containing 6.6% formaldehyde and, finally, blotted onto nytran membranes. Poly A RNA was prepared by passing the total RNA through oligo dT cellulose columns.

*Probes*

The 2.5 kb *Bam*H1 fragment of the human pHp53B cDNA (ATCC) was used for analysis of the p53 gene and its expression. This fragment contains 135 bp upstream of the first ATG, the entire coding sequence and the untranslated 3′ sequences. This probe hybridised to exons 2–11 of the mouse p53 gene. The plasmid pMSVp53G, which contains the full length murine gene (Eliyahu *et al.*, 1984), was used for structural analysis of the mouse p53 gene. For analysis of the 5′ end of the gene a 0.73 kb *Hind*III/*ECo*R1 fragment was excised from pMSVp53G and cloned into a puc 19 vector. For analysis of the human first exon we used plasmid pBT53 (Lamb & Crawford, 1986). The p 4.7R human Rb cDNA (Friend *et al.*, 1987) was cut to release the 3.8 and 0.6 kb segments to study the 3′ and 5′ regions of the gene, respectively. The murine Rb cDNA (pmRb102) (Bernards *et al.*, 1989) was employed for studies of the murine gene. The rat β-actin (pR βA-1) was obtained from the laboratory of Dr Lawrence Kedes and used as a control in RNA hybridisations. All inserts were labelled by random priming method (Feinberg & Vogelstein, 1983).

*Hybridisation*

The hybridisation and washing were performed under high stringency conditions using 50% formamide, 5 × SSC, 5 × Denhardt's solution, 0.02 M phosphate buffer, and 50 mg ml$^{-1}$ carrier DNA at 42°C. Wherever human probes were used for analysis of mouse DNA, the hybridisations were carried out at 37°C.

*Densitometric measurements*

Measurements of autoradiographs were carried out using a Molecular Dynamics computing densitometer.

### Results

*Rearrangement of p53 gene in murine osteosarcoma*

The C3HOS radiation-induced osteosarcoma was converted into a permanent culture line and subsequently cloned in our laboratory (see methods). Two tumourigenic clones, B10 and F6, were chosen for further analysis. Upon inoculation into C3H/F/Sed animals, F6 produces highly differentiated osteosarcomas containing abundant calcified osteoid, while the B10 clone produces poorly differentiated tumours with minimal calcification. Figure 1 shows the histological appearance of the two tumours obtained 4–6 weeks after subcutaneous injection of 1 × 10$^6$ cells.

The structure of the p53 gene was examined in the DNA samples obtained from the F6 and B10 tumours, and MC3T3-E1 osteoblast cells. The MC3T3-E1 cells were derived from a C57 mouse, but no DNA polymorphisms have been associated with the chromosomal region under study (Zakut-Houri *et al.*, 1983). Digestion of the control DNA specimens with *Eco*R1 generated a 16 kb fragment containing a b

**Figure 1** Histology (haematoxylin and eosin) of the mouse C3HOS tumour. **a**, Clone F6 is a well differentiated variant presenting abundant osteoid; **b**, clone B10 resembles undifferentiated soft tissue sarcoma with little osteoid.

the functional p53 gene and a 3.3 kb fragment containing the pseudogene (Zakut-Houri *et al.*, 1983) (Figure 2). Both F6 and B10 tumour DNA retained an intact pseudogene but contained a truncated 13 kb p53 gene. Faint traces of a 16 kb fragment appearing on Southern blots of tumour DNA are of stromal origin. This was confirmed when cultured F6 and B10 cells were used in place of tumour tissue. In such preparations only rearranged bands are detected, as evidenced in *Bam*H1 digests shown in Figure 2. Other restriction enzymes were employed in efforts to produce fragments that cut within the p53 gene. The enzymes, *Hind*III and *Bam*H1, cut in the 6.1 kb first intron region as well as at other sites of the gene (Figure 2). Other enzymes, *Sac*-I, *Kpn*-I and *Pst*-I also cut within this region (Bienz *et al.*, 1984) and were therefore employed. *Bam*H1 digestion gave 10.5 (pseudogene) and 6.0 kb bands in normal DNA. Part of the first intron and exons 2–6 of the p53 gene are contained within the 6.0 kb fragment. This fragment appeared as a 7.5 kb band in the tumours. Similar results were observed with *Hind*III. Data obtained with other restriction enzymes are shown in Table I. Digestions of control and tumour DNA with *Sac*-I or *Pst*-I produced normal band patterns. In contrast, *Kpn*-1 digestion of DNA from F6 and B10 tumours gave a 6.0 kb band in place of a 4.5 kb fragment obtained from control cells (Table I). It appeared that the rearrangement occurred 5′ to the second exon and is located within the intron. The p53 gene was similarly analysed in the original primary tumour as well as the other cell clones. In all cases, the rearrangements within the p53 gene were identical to those described for F6 and B10 tumours (data not shown).

In order to further define the location and nature of

**Figure 2** The analysis of the p53 gene in mouse osteosarcoma. High molecular DNA from solid tumours and/or cultured cells was digested with indicated restriction enzymes and hybridised to the cDNA probe php53B. A map of murine p53 gene shows the restriction enzyme sites on the gene. (Bienz *et al.*, 1984).

**Table I** p53 restriction fragments in normal and transformed cells

| | | Size of restriction fragments (kb) | | |
|---|---|---|---|---|
| *Cell line/tumour* | *P53 producer* | *Sac-1* | *Pst-1* | *Kpn-1* |
| MC3T3-E1 | + | 13.5, 5.0, 2.5, 0.6 | 3.0, 1.4, 1.2, 0.4 | 11, 4.5, 2.3, 1.25 |
| C3HF6 tumour | – | 13.5, 5.0, 2.5, 0.6 | 3.0, 1.4, 1.2, 0.4 | 11, 6.0, 2.3, 1.25 |
| C3HB10 tumour | – | 13.5, 5.0, 2.5, 0.6 | 3.0, 1.4, 1.2, 0.4 | 11, 6.0, 2.3, 1.25 |

Southern Blot analysis was performed as described under Methods and hybridised to the cDNA probe php53B. Values represent the approximate size of hybridising DNA fragments obtained with respective restriction enzyme. The fragment sizes were calculated by comparing their relative migrations with that of *Hind*III digested λ DNA and *Hae*III digested φX174 DNA.

alteration, we utilised genomic DNA probes to determine the status of the non-coding first exon. The mouse probe specific for the first exon when hybridised to the ECoRI-digested DNA identified a novel 3.8 kb fragment, but did not bind to the larger 13 kb fragment (Figure 3). In control tissue, this probe bound to the 16 kb fragment. In *Kpn*-I digests a 6.0 kb band was seen in place of a 5.2 kb band (Figure 3). The same was true for the *Sac*-I digest. In normal tissue, *Sac*-I cuts at sites in intron-1 and 5′ to exon-1 yielding a 3.7 kb fragment. In tumours, a fragment about 0.6 kb larger than normal was obtained (data not shown). The above data demonstrate that the rearrangement was contained within the first 3 kb of the p53 first intron. The appearance of a novel fragment within the *ECo*R1 digest, about 0.8 kb larger than the expected length, suggested the presence of a sequence integrated within the first intron region. Further characterisation of this region will be required to confirm the nature of this integrated sequence. The integrated DNA, however, did not seem to markedly alter the region in its immediate vicinity and, therefore, most other restriction sites appeared intact.

*Northern analysis*

The transcription of the p53 gene was analysed by means of Northern analysis of total RNA isolated from tumour and control cells. While a normal 2.0 kb transcript was present in osteoblast cells, no message could be detected in the tumours (Figure 4). In order to improve sensitivity, the poly A mRNA was used in place of total RNA preparations. Five μg of poly A mRNA was run on a gel and blotted, but no p53 message could be detected. All Northern blots were rehybridised to β-actin for control and quantitative purposes utilising a 2.2 kb β-actin message (Figure 4).

*Structure of p53 gene in human osteosarcomas*

Five osteosarcoma cell lines that were available in our laboratory were screened for structural and expression characteristics of their p53 gene using *ECO*R1 and *Hind*III digests. As seen from the map (Figure 5), *ECO*R1 cuts the gene into two fragments, the smaller one carrying the first

**Figure 3** Analysis of p53 genomic DNA fragments detailing the first exon region. DNA aliquots from MC3T3 E − 1 osteoblast cells, an early passage of the C3HOS original tumour (C3H tumour), B10 and G8 clones were digested with *ECO*R1 and hybridised to a mouse p53 first exon specific probe. The same analysis was also carried out in Kpn-I digested DNA.

**Figure 4** Expression of p53 gene in mouse osteosarcoma. Total RNA was separated, blotted and hybridised to php53 **a**. The same blot rehybridised to $\beta$ − actin **b**. Analysis with poly A − RNA **c**.

**Figure 5** **a** Southern analysis of p53 gene in human osteosarcoma cells. Equal amounts of DNA from the various cell lines were cut with *ECO*R1 or *Hind*III and hybridised to a human p53 cDNA probe php53B. The same blot was rehybridised with a probe for $\alpha$ − actin. **b**, Northern blot analysis of total RNA with php53B probe for steady state message (upper panel). The same blot was rehybridised to $\beta$ − actin (lower panel).

exon region and a larger piece containing the rest of the gene. This analysis provided us with information about the status of the first exon as well. The status of the first exon was also confirmed by rehybridising the blot with the human first exon probe pBT53. This probe hybridised to a 3.7 kb fragment in all cell lines (data not shown). For densitometric measurement and to check variations in DNA concentrations, the blot was also hybridised to $\alpha$-actin. On hybridisation to the php53B probe, the expected bands (15.0 and 3.7 kb) were obtained in *ECO*R1 digests of the control cells. However, the same analysis of cultured osteosarcoma cells revealed notable differences. G-292 and MG-63 osteosarcoma contains a 10 kb band in addition to the 15 kb band (Figure 5). Both of these cell lines (MG-63 and G-292), therefore, seem to contain rearrangement in the first intron of the p53 gene, which is about 10 kb long in humans (Masuda *et al*., 1987). A normal band pattern was seen with the HOS cell cell line although with an increased intensity, but densitometric measurements did not reveal any significant amplification of the gene. Saos-2 lost most of the gene, with the exception of the first exon, as documented by its hybridisation to the 3.7 kb band. This finding was interesting, as it suggested that the deletion was not random but had occurred in the intron region. Of all osteosarcomas tested, only U-2OS cells presented a normal hybridising pattern, although much weaker in its intensity, as determined by densitometry. Similar analyses were conducted with *Hind*III digests, which resulted in two bands of 7.0 and 2.5 kb (Figure 5a), both of which represent fragments originating from the middle and 3′ end of the gene and, therefore, not informative for alterations in the first intron region. Decreased hybridisation was documented for MG-63 and U-2OS cells. Densitometry confirmed that MG-63 contained only one allele which was rearranged, while U-2OS had only one allele which appeared structurally intact. Saos-2 cells showed no p53 probe hybridising bands (Figure 5a).

*Northern analysis of human p53 gene*

p53 was transcribed into a 2.3 kb message in all control tissues (Figure 5b). Most of the osteosarcoma cell lines do not contain any transcript (G-292, Saos-2, and MG-63), and only HOS cells showed increased levels of p53 message. Even though U-2OS had only one allele for p53, it appeared to make normal amounts of the p53 transcript (Figure 5b).

*Analysis of Rb gene in osteosarcoma*

We screened for changes within the Rb gene in human osteosarcoma cell lines, as well as in the mouse tumour to determine if inactivation of Rb was a common event in these tumours. No gross alterations were visible on Southern blots in the DNA from the murine C3HOS tumours (Figure 6). The presence of a normal 4.7 kb transcript could be detected in the osteoblast cell line MC3T3-E1, as well as in tumours. A similar analysis was conducted with human osteosarcoma cell lines. The 3.8 and 0.6 kb Rb gene specific probes span a large region of the Rb gene and bind to several sequences on *Hind*III digests as depicted in Figure 7. In *Hind*III digests, deletion of the 3′ end of the Saos-2 gene was evident. U-2OS showed decreased hybridisation to these probes, but no quantitative differences were found. The latter alterations were confirmed with *ECO*R1 digests (not shown). Hybridisation of the 0.6 kb probe to *Hind*III digest showed that 14.0, 7.0, 6.0, 1.5, and 1.2 kb fragments were present in all the osteosarcoma cell lines tested. U-2OS cells are likely to contain only one intact allele as judged by decreased hybridisation. In the Y79 retinoblastoma cell line, the 7.0 kb band was absent. Northern analysis was performed with the 3.8 kb probe and 4.7 kb transcripts could be detected in all cell lines, with the exception of the Saos-2 and Y79 cell lines (data not shown).

*Analysis of c-*myc *in the murine tumour*

We also attempted to determine whether C3HOS contained tumour alterations in c-*myc*, as this has been a consistent observation in murine osteosarcoma tumours (Sturm *et al*., 1990). Assays were conducted to uncover alterations in c-*myc* gene structure and expression. *ECO*R1 digests of normal and tumour DNA gave a 20 kb band for c-*myc* gene in all the samples analysed and expression of normal levels of a 2.2 kb RNA transcript was observed (data not shown).

### Discussion

A large array of mutations affecting the p53 gene have been identified and are thought to play a role in the transformation of not only skeletal, but also many other types of cells (Levine *et al*., 1991; Hollstein *et al*., 1991). Our data are in agreement with the results of Masuda *et al*. (1987) and

**Figure 6** The Rb gene in mouse osteosarcoma. **a**, High molecular weight DNA from C3H mouse liver (control), C3HOS, F6 and B10 tumours was digested with *Hind*III, electrophoresed and blotted. Hybridisations were done with a murine Rb cDNA probe pRB102. **b**, Northern analysis of Rb gene expression. Total RNA isolated and blotted as described in text was hybridised to the probe pRB102.

Mulligan *et al*. (1990) and indicate that inactivation of p53 is a common event in osteosarcoma development. The exact role of the p53 gene either in the transformation process, or in normal functions of bone cells is not known. The rearrangement identified in the C3HOS murine osteosarcoma involves the first intron. Interestingly, the same lesion was found in three out of five human tumour cell lines. In comparing the two anti-oncogenes, the structural rearrangement of the p53 gene is more prevalent than similar aberrations of the Rb gene in the cell lines studied. Four of the five human osteosarcoma cell lines showed aberrant expression of p53. Among these, MG-63 and G-292 show gene rearrangement in the first intron, while Saos-2 retained only the region harbouring first exon and lost the rest of the gene. It is therefore possible that an alteration in the first intron was responsible for the resultant changes in Saos-2 cells. Rearrangement of the p53 gene in osteosarcoma cell lines has been previously observed, and our data on MG-63 generally agree with the published reports (Masuda *et al*., 1987; Miller *et al*., 1990). Our data concerning p53 expression in G-292 are in agreement with observations by Diller *et al*. (1990), but differ from those reported by Miller *et al*. (1990), who reported synthesis of p53 RNA in these cells. It is not clear why there is a discrepancy regarding the expression of p53 in G292. HOS is known to harbour a point mutation within the p53 coding sequence and overproduces a mutant p53 product (Romano *et al*., 1989).

The rearrangement of p53 in murine C3HOS osteosarcoma

**Figure 7** Rb gene in human osteosarcoma cell lines. High molecular weight DNA was cut with *Hin*dIII and blotted and hybridised to Rb probes (**a** & **b**). Two human RB probes spanning the whole gene, p3.8 and p0.6 were used for analysis of the whole gene and are indicated at the bottom of the figure with the *Hin*dIII fragments they bind to (**c**).

is present in the original radiation-induced tumour, as well as its clonal derivatives. The clones produce tumours with different growth rates and properties upon injection into animals. The rearrangement within the p53 gene has not changed over many generations both *in vitro* and *in vivo*. This indicates the importance of the described p53 aberration for the maintenance of the tumourigenic state. This is further supported by a recent study of primary osteosarcomas where 18% of the tumours that had rearranged p53 genes, had the alteration mapping to the first intron region (Miller *et al.*, 1990). The inactivation of p53 via alteration within the first intron thus appears to be unique to osteosarcoma and different from other human cancers where p53 inactivation occurs primarily by a loss of alleles and/or point mutations. This does not, however, preclude the occurrence of other mutations. In fact, in a recent study of sarcomas, gross alterations in p53 with lack of expression, or aberrant expression of p53 were detected at high frequency in osteosarcoma and rhabdomyosarcoma (Mulligan *et al.*, 1990). Irrespective of the mechanism, the alteration of normal p53 function has an important role in neoplasia.

Gross rearrangements of p53 have been reported in virus-infected erythroleukaemia cells (Mowat *et al.*, 1985; Munroe *et al.*, 1990), as well as in Ab-MuLv transformed lymphoid cells (Wolf & Rotter, 1984). In the latter case, the inactivation of the gene is a result of an insertion of the Mo-MuLV-like DNA sequences into the first intron of the active gene in these cells (Wolf & Rotter, 1984). These modifications appear analogous to those seen in osteosarcomas and may reflect a common pathway for tumour initiation from multipotent precursor cells. Alternately, its similarity to virus-induced alterations suggest that activation of endogenous retroviruses that frequently occur in radiation-induced tumours may have a role in the induction of the murine tumour (Schmidt *et al.*, 1988).

Amplification of the c-*myc* gene has been reported in 30% of the murine osteosarcomas tested (Strum *et al.*, 1990). We did not see amplification of c-*myc* in the C3HOS tumour or the human osteosarcoma cell lines. The absence of the oncogene amplification phenomenon could be due to the fact that this change is a progression-related event, not directly involving the carcinogenic process.

Our studies showed that structural rearrangements of Rb antioncogenes may not be prevalent in human osteosarcoma, since only one out of five cell lines showed gross alteration of the Rb gene. The Rb gene in the mouse tumour also appears intact and synthesises a normal 4.7 kb transcript. However, given the large size of the gene and the wide variety of ways that the Rb gene can be inactivated (Cavenee, 1991), our data cannot exclude the involvement of Rb in neoplastic transformation of cells under study. Nevertheless, the analyses of Rb protein have been carried out in the same osteosarcoma cell lines we examined and have appeared normal (Shew *et al.*, 1989).

A large amount of data have been accumulated concerning the various p53 mutations in human cancers (Levine *et al.*, 1991; Hollstein *et al.*, 1991). Functionally, p53 appears similar to the retinoblastoma gene (Levine *et al.*, 1991). It, therefore, seems reasonable to suggest that the tumourigenic process may require inactivation of either one of the genes. Alternatively, if p53 and Rb antioncogenes form a part of the cellular interactive regulatory mechanism, inactivation of one may, in effect, lead to inactivation of the other. Intron functions of several genes in transgenic mice have been tested (Brinster *et al.*, 1988), and were found to increase the transcriptional efficiency of genes introduced into mice. This observation has also been extended to include the p53 gene (Lozano *et al.*, 1991). Studies are in progress to determine the nature of alteration in the first intron of the human and murine osteosarcoma p53 genes.

The authors wish to thank Moshe Oren for pMSVp53G plasmid, Thaddeus Dryja for the p4.7R Rb cDNA, Rene Bernard for pmRb102, Simon Tuck and Lionel Crawford for pBT53, and Lawrence Kedes for β actin plasmid. The authors also thank Ms Teresa Hentosz for her technical assistance with tissue culture and Ms Laurie Iannuzzi for her help with the preparation of this manuscript.

### References

BAKER, S.J., FEARSON, E.R., NIGRO, J.M. & 9 others (1989). Chromosome 17 deletions and p53 gene mutations in colorectal carcinomas. *Science*, **244**, 217.

BERNARDS, R., SCHACKLEFORD, G.M., GERBER, M.R. & 8 others (1989). Structure and expression of the murine retinoblastoma gene and characterization of its encoded protein. *Proc. Natl Acad. Sci. USA*, **86**, 6474.

BIENZ, B., ZAKUT-HOURI, R., GIVOL, D. & OREN, M. (1984). Analysis of the gene coding for the murine cellular tumour antigen p53. *EMBO J.*, **3**, 2179.

BRINSTER, R.L., ALLEN, J.M, BEHRINGER, R.R., GELINAS, R.E. & PALMITER, D. (1988). Introns increase transcriptional efficiency in transgenic mice. *Proc. Natl Acad. Sci. USA*, **85**, 836.

CAVENEE, W.K. (1991). Recessive mutations in the causation of human cancer. *Cancer*, **67**, 2431.

CHANDAR, N., LOMBARDI, B., SCHULZ, W. & LOCKER, J. (1987). Analysis of *Ras* genes and linked viral sequences in rat hepatocarcinogenesis. *Am. J. Pathol.*, **129**, 232.

CHOI, C.H., SEDLACEK, R.S. & SUIT, H.D. (1979). Radiation-induced osteogenic sarcoma of C3H mouse. *Eur. J. Cancer*, **15**, 433.

CHOMCZYNSKI, P. & SACCHI, N. (1987). RNA extraction with guanidium isothioscynanate. *Anal. Biochem.*, **162**, 156.

DRYJA, T.P., RAPAPORT, J.M., EPSTEIN, J. & 4 others (1986). Chromosome 13 homozygosity in osteosarcoma without retinoblastoma. *Am. J. Hum. Genet.*, **38**, 59.

DILLER, L., KASSEL, J., NELSON, C.E. & 8 others (1990). P53 functions as a cell cycle control protein in osteosarcoma. *Mol. Cell. Biol.*, **10**, 5772.

ELIYAHU, D., RAS, A., GRUSS, P., GIVOL, D. & OREN, M. (1984). Participation of p53 cellular tumor antigen in transformation of normal embryonic cells. *Nature*, **312**, 646.

ELIYAHU, D., MICHALOVITZ, D., ELIYAHU, S., PINHASI-KIMHI, O. & OREN, M. (1989). Wild-type p53 can inhibit oncogene-mediated focus formation. *Proc. Natl Acad. Sci. USA*, **86**, 8763.

FEINBERG, A.P. & VOGELSTEIN, B. (1983). A technique for radiolabeling DNA restriction endonuclease fragments to high specific activity. *Anal. Biochem.*, **132**, 6.

FINLAY, C.A., HINDS, P.W. & LEVINE, A.J. (1989). The p53 protooncogene can act as a suppressor of transformation. *Cell*, **57**, 1083.

FRIEND, S.H., BERNARDS, R., ROGELJ, S. & 4 others (1987). A human DNA segment with properties of the gene that predisposes to retinoblastoma and osteosarcoma. *Nature*, **323**, 643.

GILMAN, P.A., WANG, N., FAN, S.-F., REEDE, J., KHAN, A. & LEVENTHAL, B.G. (1985). Familial osteosarcoma associated with 13;14 chromosomal rearrangement. *Cancer Genet. & Cytogenet.*, **17**, 123.

HANSEN, M.F., KOUFOS, A., GALLIE, B.L. & 5 others (1985). Osteosarcoma and retinoblastoma: a shared chromosomal mechanism revealing recessive predisposition. *Proc. Natl Acad. Sci, USA*, **82**, 6126.

HARBOUR, J.W., LAI, S.-L., WHANG-PENG, J., GAZDAR, A.F., MINNA, J.D. & KAYE, F.J. (1988). Abnormalities in structure and expression of the human retinoblastoma gene in SCLC. *Science*, **241**, 353.

HOLLSTEIN, M., SIDRANSKY, D., VOGELSTEIN, B. & HARRIS, C.C. (1991). p53 mutations in human cancers. *Science*, **253**, 49.

IKEDA, S., SUMIJ, J., AKIYAMA, K. & 6 others (1989). Amplification of both c-*myc* and c-*raf*-1 oncogenes in human osteosarcoma. *Jpn. J. Cancer Res.*, **80**, 6.

KODAMA, H., AMAGAI, Y., SUDO, H., KASAI, S. & YAMAMOTO, S. (1981). Establishment of a clonal osteogenic cell line from new born mouse calvaria. *Jpn. J. Oral Biol.*, **23**, 899.

LAMB, P. & CRAWFORD, L. (1986). Characterization of the human p53 gene. *Mol. Cell. Biol.*, **6**, 1379.

LEE, W.-H., BOOKSTEIN, R., HONG, F., YOUNG, L.-J., SHEW, J.-Y. & LEE, E.Y.-H. (1987). Human retinoblastoma susceptibility gene: cloning, identification, and sequence. *Science*, **235**, 1394.

LEE, E., Y.-H.P., TO, H., SHEW, J.-Y., BOOKSTEIN, R., SCULLY, P. & LEE, W.-H. (1988). Inactivation of the retinoblastoma susceptibility gene in human breast cancers. *Science*, **241**, 218.

LEVINE, A.J., MOMAND, J. & FINLAY, C.A. (1991). The p53 tumor suppressor gene: *Nature*, **351**, 453.

LOZANO, G. & LEVINE, A.J. (1991). Tissue specific expression of p53 in transgenic mice is regulated by intron sequences. *Mol. Carcinogen.*, **4**, 3.

MASUDA, H., MILLER, C., KOEFFLER, H.P., BATTIFORA, H. & CLINE, M. (1987). Rearrangement of the p53 gene in human osteogenic sarcoma. *Proc. Natl Acad. Sci. USA*, **84**, 7716.

MILLER, C., MOHANDAS, T., WOLF, D., PROKOCIMER. M., ROTTER, V. & KOEFFLER, H.P. (1986). Human p53 gene localized to short arm of chromosome 17. *Nature*, **319**, 783.

MILLER, C., ASLO, A., TSAY, C. & 6 others (1990). Frequency and structure of p53 rearrangements in human osteosarcoma. *Cancer Res.*, **50**, 7950.

MOWAT, M., CHENG, A., KIMURA, N., BERNSTEIN, A. & BECHIMOL, S. (1985). Rearrangements of the cellular p53 gene in erythroleukaemic cells transformed by Friend virus. *Nature*, **314**, 633.

MULLIGAN, L.M., MATLASHEWSKI, G.J., SCRABLE, H.J. & CAVENEE, W.K. (1990). Mechanisms of p53 loss in human sarcomas. *Proc. Natl Acad. Sci. USA*, **87**, 5863.

MUNROE, D.G., PEACOCK, J.W. & BENCHIMOL, S. (1990). Inactivation of the cellular p53 gene is a common feature of friend virus-induced erythroleukemia: relationship of inactivation to dominant transforming alleles. *Mol. Cell Biol.*, **10**, 3307.

NIGRO, J.M., BAKER, S.J., PREISINGER, A.C. & 13 others (1989). Mutations in the p53 gene occur in diverse human tumor types. *Nature*, **342**, 705.

REISSMANN, P.T., SIMON, M.A.., LEE, W.-H. & SLAMON, D.J. (1989). Studies of the retinoblastoma gene in human sarcomas. *Oncogene*, **4**, 839.

ROMANO, J.W., EHRHART, J.C., DUTHU, A., KIM, C.M., APELLA, E. & MAY, P. (1989). Identification and characterization of a p53 gene mutation in a human osteosarcoma cell line. *Oncogene*, **4**, 1483.

SCHMIDT, J., LUZ, A. & ERFLE, V. (1988). Endogenous murine leukemia viruses: frequency of radiation-activation and novel pathogenic effects of viral isolates. *Leukemia Res.*, **12**, 393.

SCHON, A., MICHIELS, L., JANOWSKI, M., MERREGAERT, J. & ERFLE, V. (1986). Expression of protooncogenes in murine osteosarcomas. *Int. J. Cancer*, **38**, 67.

SHEW, J.-Y., LING, N., YAND, X., FODSTAD, O. & LEE, W.-H. (1989). Antibodies detecting abnormalities of the retinoblastoma susceptibility gene product ($pp110^{Rb}$) in osteosarcomas and synovial sarcomas. *Oncogene Res.*, **1**, 205.

STURM, S.A., STRAUSS, P.G., ADOLPH, S., HORST, H. & VOLKER, E. (1990). Amplification and rearrangement of c-*myc* in radiation-induced murine osteosarcomas. *Cancer Res.*, **50**, 4146.

TOGUCHIDA, J., ISHIZAKE, K., SASAKI, M.S. & 4 others (1988). Chromosomal reorganization for the expression of recessive mutation of retinoblastoma susceptibility gene in the development of osteosarcoma. *Cancer Res.*, **48**, 3939.

TOGUCHIDA, J., ISHIZAKI, K., NAKAMURA, Y. & 6 others (1989). Assignment of common allele loss in osteosarcoma to the subregion 17p13. *Cancer Res.*, **49**, 6247.

WOLF, D. & ROTTER, V. (1984). Inactivation of p53 Gene Expression by an Insertion of Moloney Murine Leukemia Virus-Like DNA Sequences. *Mol. Cell Biol.*, **4**, 1402.

WU, J.-X., CARPENTER, P.M., GRESSENS, C. & 4 others (1990). The proto-oncogene c-fos is overexpressed in the majority of human osteosarcomas. *Oncogene*, **5**, 989.

ZAKUT-HOURI, R., OREN, M., BIENZ, B., LAVIE, B., SAZUM, S. & GIVOL, D. (1983). A single gene and a pseudogene for the cellular tumor antigen p53. *Nature*, **306**, 594.